# LPC18:0 Secreted by Exogenous Neural Stem Cells Potentiates Neurogenesis and Functional Recovery via GPR55‐Mediated Signalling in Spinal Cord Injury

**DOI:** 10.1111/cpr.70146

**Published:** 2025-11-16

**Authors:** Dong Chen, Shuo Liu, Le‐Yi Tu, Ming‐Mei Yang, Cong‐Wang Xu, Yue Jiang, Hui Yang, Chen‐Xu Tai, Yan‐Ning Wang, Yuan‐Yuan Xie, Ping‐Ping Shen, Bin Wang

**Affiliations:** ^1^ Clinical Stem Cell Center, Nanjing Drum Tower Hospital, Affiliated Hospital of Medical School Nanjing University Nanjing Jiangsu China; ^2^ State Key Laboratory of Pharmaceutical Biotechnology and Department of Urology, Nanjing Drum Tower Hospital, The Affiliated Hospital of Nanjing University Medical School, School of Life Sciences Nanjing University Nanjing Jiangsu China; ^3^ Clinical Stem Cell Center, Nanjing Drum Tower Hospital, Clinical Medical College of Traditional Chinese and Western Medicine Nanjing University of Chinese Medicine Nanjing Jiangsu China; ^4^ China Hospital Reform and Development Research Institute of Nanjing University, Nanjing Drum Tower Hospital, Affiliated Hospital of Medical School Nanjing University Nanjing Jiangsu China

## Abstract

Spinal cord injury (SCI) is a devastating condition with limited therapeutic options. Although neural stem cell (NSC) transplantation shows regenerative potential, its efficacy is constrained by the hostile post‐injury microenvironment. Here, we employed untargeted metabolomics to investigate metabolic reprogramming induced by NSC‐loaded multichannel collagen scaffolds in a rat SCI model. NSC transplantation significantly enhanced functional recovery and structural remodelling, concomitant with elevated neurogenesis and attenuated gliosis. Metabolomic profiling identified lysophosphatidylcholine 18:0 (LPC18:0) as a key NSC‐derived metabolite. Mechanistically, LPC18:0 promoted the differentiation of endogenous NSCs into neurons via the GPR55/AKT/GSK3β signalling axis, as validated by receptor‐specific inhibition. In vivo administration of LPC18:0 improved motor function, axonal regeneration and recruitment of immature neurons. These findings reveal a novel metabolic mechanism underlying NSC‐based therapy, positioning LPC18:0/GPR55/AKT/GSK3β signalling as a therapeutic target for SCI recovery.

## Graphical abstract

LPC18:0 secreted by exogenous neural stem cells potentiates neurogenesis via the GPR55/AKT/GSK3β signalling axis, which ultimately promotes spinal cord injury recovery.

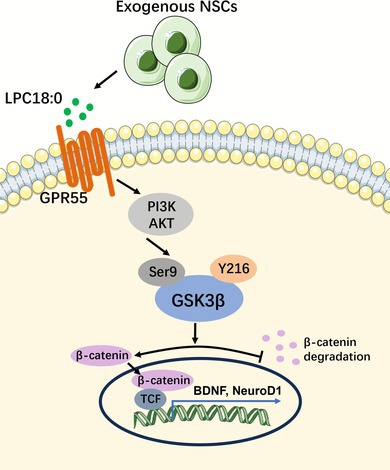

## 1 Introduction

Spinal cord injury (SCI) represents a global health challenge and a leading cause of permanent disability and mortality. Traumatic spinal cord injuries constitute the most prevalent clinical manifestation [1]. Following primary mechanical damage, secondary pathological events, including neuronal apoptosis, glial scar formation, demyelination, excitotoxicity and proinflammatory factor release, exacerbate neurological dysfunction and tissue damage [2]. Consequently, current clinical interventions, such as early surgical decompression, hemodynamic stabilisation and high‐dose methylprednisolone administration, have demonstrated limited efficacy in promoting tissue regeneration and functional recovery [3].

Advances in stem cell‐based tissue engineering have yielded promising therapeutic strategies for injured spinal cord repair [4]. Neural stem cells (NSCs), as tissue‐specific cells within the central nervous system, have garnered significant attention because of their high self‐renewal capacity and trilineage differentiation potential. Substantial preclinical and clinical evidence indicates that NSC transplantation confers structural and functional benefits following SCI [5, 6, 7]. However, the reparative efficacy of NSCs is substantially constrained by the dysregulated post‐injury microenvironment, which impedes sustained graft survival and functional integration [8]. Notably, within the SCI milieu, transplanted NSCs exhibit preferential differentiation towards astrocytes rather than towards neurons or oligodendrocytes [9, 10]. Increasing evidence suggests that the therapeutic mechanisms of NSC transplantation extend far beyond simple replacement of damaged cells, encompassing neuroprotective effects, immunomodulation and facilitation of axonal regeneration [5, 6]. Nonetheless, the functional significance and underlying molecular mechanisms of grafted NSCs in SCI remain incompletely characterised, particularly with respect to metabolic pathway regulation.

It has been reported that SCI leads to multiple metabolic disturbances, including dysregulated glycolysis, aberrant lipid metabolism and mitochondrial dysfunction [11, 12]. These metabolic disorders cause further pathological progression at the injury site, such as glial scar formation, neuronal cell death and neuroinflammation, and eventually lead to the failure of SCI recovery [13, 14]. Recent developments in metabolomics technology have attracted significant interest because of their potential to associate small‐molecule metabolites with disease progression on the basis of high‐throughput mass spectrometry analysis. As terminal products or intermediates of biological processes, small‐molecule metabolites not only serve as biomarkers reflecting the functional status of biological systems but also regulate neuroinflammation and the microenvironment following SCI [15, 16]. For example, the physiological concentrations of LPCs are closely associated with nerve damage. The most abundant LPC species, LPC16:0, is increased in brain tissue after traumatic brain injury, whereas all other detected LPC species exhibit decreasing trends [17]. Another study revealed that the levels of LPC16:0, LPC18:0 and LPC18:1 in the spinal cord rapidly increase to comparable concentrations within 75 min following partial sciatic nerve injury [18]. Moreover, the concentration of LPC18:0 in the spinal cord was significantly lower than that of LPC16:0 after SCI, although both LPC16:0 and LPC18:0 concentrations tended to increase [19]. In addition, small‐molecule metabolites can directly bind to target proteins to modulate disease‐associated signalling pathways, thereby regulating physiological functions and influencing disease progression [20, 21]. As shown by Quan et al., LPC18:0 enhances macrophage phagocytosis by activating the AMPK/p38 MAPK pathway, suggesting its potential as a treatment for bacterial infections [22].

In view of the above findings, characterising spatiotemporal alterations in metabolic profiles within the injured spinal cord following NSC transplantation represents a strategic approach for therapeutic development, although the metabolic reprogramming mediated by exogenous NSCs in SCI treatment remains unexplored. Our prior study established a biologically compatible multichannel collagen scaffold that facilitates the sustained retention of transplanted NSCs at the site of SCI [23]. In the present study, to comprehensively evaluate the systemic impact of grafted NSCs within the post‐injury microenvironment, we performed global metabolomic profiling of lesioned injured spinal cord tissue integrated with bioinformatics and statistical analysis. Subsequently, functional validation and mechanistic investigations of candidate metabolites were performed through in vitro and in vivo experimental paradigms. Overall, our findings highlight the pivotal regulatory role of NSC‐derived metabolites in the post‐injury microenvironment, offering novel mechanistic insights into metabolic reprogramming for SCI recovery via cell‐based therapeutics.

## 2 Methods

### 2.1 Rat NSC Culture and Differentiation

Primary NSCs were obtained following a previous report [23]. Briefly, hippocampi dissected from embryonic day 14.5 (E14.5) Sprague–Dawley (SD) rat embryos were digested with Accutase (A6964, Merck) at 37°C for 20 min. Following washing with 0.01 M PBS, the tissues were triturated via a pipette and filtered through a 40 μm cell strainer to generate a single‐cell suspension. Single NSCs were cultured in complete DMEM‐F12 medium (10565018, Gibco) supplemented with 20 ng/mL epidermal growth factor (EGF, 315‐09, Thermo Fisher Scientific), 20 ng/mL basic fibroblast growth factor (bFGF, 450‐33, Thermo Fisher Scientific), 2% B27 (17504044, Gibco) and 1% penicillin–streptomycin (15070063, Gibco). After 3–4 days of culture under proliferative conditions, free‐floating neurospheres formed and were harvested for subsequent experiments. NSC neurosphere identity was confirmed by immunostaining with an anti‐Nestin antibody, a classical NSC marker.

For differentiation assays, dissociated single NSCs were plated onto poly‐d‐lysine‐coated glass coverslips and maintained in differentiation‐inducing medium (DMEM‐F12 medium, 2% B27 and 1% penicillin–streptomycin) for 5 days. Cell differentiation into neurons and astrocytes was assessed by immunofluorescence using mouse monoclonal anti‐βIII‐tubulin (Tuj‐1; ab7751; Abcam; 1:500) and rabbit polyclonal anti‐glial fibrillary acidic protein (GFAP; ab7260; Abcam; 1:500), respectively.

### 2.2 Scanning Electron Microscopy (SEM)

The multichannel collagen scaffolds were dehydrated through a gradient ethanol series (50%, 75%, 95% and 100% v/v) and lyophilised. Fully freeze‐dried collagen scaffolds were sputter‐coated with a 10‐nm platinum layer and imaged via SEM (S‐3400N, Hitachi).

### 2.3 SCI Model and Experimental Design

Animal surgical procedures were approved by the Animal Research Ethics Committee of the Affiliated Drum Tower Hospital of Nanjing University Medical School and were performed in accordance with the National Institutes of Health guidelines. Adult female SD rats (weighing 220–250 g), obtained from the Laboratory Animal Center of Nanjing Medical University, were housed in specific pathogen‐free (SPF) facilities under controlled temperature and humidity conditions with a 12‐h light/dark cycle for 10 days prior to surgery.

The rats were anaesthetised with isoflurane. The dorsal skin overlying the surgical site was shaved and disinfected with povidone‐iodine solution. The rat SCI model was established as follows: A laminectomy was performed at vertebral levels T7–T9 to expose the spinal cord. Then, a 3‐mm‐long segment of spinal cord tissue was completely transected. Sham‐operated rats received a laminectomy only. For cell transplantation, NSCs (1 × 10^6^ cells in 20 μL of normal saline) were loaded into a presterilised multichannel collagen scaffold (3 × 3 × 3 mm) and engrafted into the cavity at the injury site.

Stock solutions (10 mM) of LPC18:0 and sphingosine were prepared in water and dimethyl sulfoxide (DMSO), respectively. For drug administration, LPC18:0 and sphingosine were diluted to 10 μM in normal saline. Then, 20 μL of the diluted solution was carefully injected into the collagen scaffold, which had already been placed at the injury site. The rats in the scaffold‐only control group were implanted with a collagen scaffold loaded with 20 μL of normal saline. The subcutaneous tissue and skin were sutured following haemostasis. Post‐operative care included the administration of ceftriaxone sodium and manual bladder expression twice daily.

### 2.4 Behavioural Evaluation

Hindlimb locomotor function in all experimental rats was quantitatively assessed via the Basso–Beattie–Bresnahan (BBB) locomotor rating scale (ranging from 0 to 21 points). BBB scores were evaluated weekly in an open field by two independent observers who were blinded to the experimental groups.

Footprint analysis was performed as previously described [24] to assess motor function recovery. The forelimbs and hindlimbs were stained with blue and red nontoxic ink, respectively. The rats were then guided to traverse a 10 cm wide × 100 cm long paper runway.

### 2.5 Untargeted Metabolomics

#### 2.5.1 Sample Preparation

Approximately 50 mg of each spinal cord tissue sample was weighed and homogenised in ice‐cold 80% methanol (1:20, tissue weight [mg]:solvent volume [μL]) via a tissue grinder. The homogenates were incubated at −40°C for 4 h and subsequently centrifuged at 13,000 × *g* for 20 min at 4°C. The supernatant was collected, dried via a centrifugal concentrator and stored at −80°C. Prior to metabolomic analysis, all the dried samples were redissolved and processed identically. A pooled quality control (QC) sample from all the spinal cord samples was prepared for monitoring the stability of the analysis. Metabolomic profiling was conducted via a Shimadzu Exion liquid chromatography system coupled with a high‐resolution time‐of‐flight mass spectrometer (SCIEX Triple TOF 5600+ System).

#### 2.5.2 Chromatographic Separation

Liquid chromatography was performed via a Shimadzu ExionLC system equipped with a Waters ACQUITY HSS T3 column (2.1 × 100 mm, 1.8 μm). The mobile phase consisted of (A) 0.1% formic acid in water and (B) 0.1% formic acid in acetonitrile. The optimised ultrahigh‐performance liquid chromatography (UHPLC) gradient elution programme was as follows: 0–1.5 min, 99.0% B; 1.5–13.0 min, 99.0%–1.0% B; 13.0–16.5 min, 1.0% B; 16.5–16.6 min, 1.0%–99% B; and 16.6–20.0 min, 99.0% B. The flow rate was maintained at 0.30 mL/min, and the column temperature was set at 40°C. A 2 μL aliquot of each sample was injected per run. The samples were analysed in a random order, with a pooled QC sample inserted after every six test samples.

#### 2.5.3 Mass Spectrometry

MS detection was performed via a Triple TOFTM 5600 MS/MS system (AB SCIEX, Foster City, USA) under the following conditions: The full scan covered a mass range from *m/z* 50 to 1000 Da. The ion spray voltage was +5500 V for positive ion mode and −4500 V for negative ion mode. Nitrogen was used as the nebuliser, heater and curtain gas at constant flow rates of 55, 55 and 35 psi, respectively. MS/MS data were acquired via an information‐dependent acquisition approach in high‐sensitivity mode with real‐time dynamic background subtraction. The collision energy was set at 35 eV with a spread of 10 eV. Mass calibration was automatically performed by the calibrant delivery system, which infused the atmospheric pressure chemical ionisation (APCI) calibration solution every five samples.

#### 2.5.4 Data Processing

Raw UHPLC‐Q/TOF‐MS data files were converted to mzXML format via MSConvert software. Peak detection, retention time alignment and feature extraction were performed via MS‐DIAL version 4.9.22128. The ion features were normalised on the basis of the total peak area of each analysed sample. Missing values were filled with one‐half of the observed minimum for that particular compound. Partial least squares discriminant analysis (PLS‐DA) was initially applied to visualise inherent metabolic differences and group separation trends. Subsequently, orthogonal PLS‐DA (OPLS‐DA) was conducted for pairwise group comparisons to identify differential ion features on the basis of variable importance in projection (VIP) values. Model validity was assessed via permutation testing (200 iterations) and cross‐validated ANOVA (CV‐ANOVA). Differential ion features were filtered using thresholds of Benjamini‐Hochberg adjusted *p* value < 0.05 and fold change (FC) > 1.5 or < 1/1.5. Metabolite identification was performed by comparing accurate mass and MS/MS fragmentation patterns against authentic standards and theoretical spectral databases. According to the Metabolomics Standards Initiative (MSI) guidelines, identification confidence was assigned at four levels: Level 1 (identified compounds) was assigned when metabolites were confirmed by comparison with an authentic standard, matching both retention time and MS/MS spectrum; Level 2 (putatively annotated compounds) was assigned for matches based on MS/MS spectral similarity with a public library; Level 3 (putatively characterised compound classes) was applied to metabolites that were only classifiable based on characteristic spectral features; and Level 4 (unknown compounds) comprised unmatched features that remained unidentified [25]. A comprehensive overview of the metabolite identification, providing MSI confidence levels, spectral match scores (dot product and reverse dot product) and associated statistical analyses (VIP, FC, *p* value), is available in Table S2.

### 2.6 Haematoxylin–Eosin (H&E) Staining

The animals were euthanised 10 weeks post‐surgery. The rats were deeply anaesthetised and transcardially perfused with 0.9% sodium chloride followed by 4% paraformaldehyde (PFA). Spinal cord segments encompassing the lesion site were dissected, postfixed in 4% PFA overnight at 4°C and cryoprotected by sequential immersion in 20% and 30% sucrose solutions. The tissues were then embedded in optimal cutting temperature (OCT) compound and sectioned coronally at 8 μm thickness via a cryostat. The sections were stained with haematoxylin, differentiated in acid alcohol (1% HCl in 70% ethanol), counterstained with eosin, dehydrated, cleared and mounted. Images were captured using an Olympus VS200 slide scanner (Olympus Corporation, Japan) and quantitatively analysed via ImageJ software.

### 2.7 Nissl Staining

Adjacent tissue sections (prepared as described above) were incubated with 1% cresyl violet solution to stain Nissl bodies specifically. Bright‐field images of Nissl‐stained sections were acquired via an Olympus VS200 slide scanner (Olympus Corporation, Japan).

### 2.8 Immunostaining

For immunofluorescence staining, the fixed tissue sections were permeabilised with 0.25% Triton X‐100 in PBS for 20 min at room temperature, followed by blocking with 5% foetal bovine serum (FBS) for 1 h. The sections were then incubated overnight at 4°C with primary antibodies diluted in blocking solution. The primary antibodies used included mouse anti‐βIII‐tubulin (Tuj‐1; ab7751; Abcam; 1:500), mouse anti‐neurofilament (NF; ab3966; Abcam; 1:500), mouse anti‐neuronal nuclei (NeuN; MA5‐33103; Thermo Fisher Scientific; 1:500), rabbit anti‐glial fibrillary acidic protein (GFAP; ab7260; Abcam; 1:500) and rabbit anti‐doublecortin (DCX; ab18723; Abcam; 1:500). After washing, the sections were incubated with appropriate Alexa Fluor‐conjugated secondary antibodies (donkey anti‐goat Alexa Fluor 488, A21202; goat anti‐mouse Alexa Fluor 568, A11031; and goat anti‐rabbit Alexa Fluor 568, A11011; all from Invitrogen; 1:1000) for 1 h at room temperature in the dark. Nuclei were counterstained with DAPI‐containing mounting medium (ab104139; Abcam). Images were acquired via confocal or fluorescence microscopy.

The immunofluorescence signal intensity for NF, NeuN and DCX at the site of injury was quantified using ImageJ software (version 1.52a). Regions of interest were defined as three randomly selected fields of view per tissue section, avoiding edges and artefacts, and the GFAP channel was used to identify injury areas. A total of three animals per group were analysed. The image files were deidentified and assigned random codes by one researcher prior to analysis. Quantitative measurements of fluorescence intensity within the defined regions of interest were then performed by another researcher who was blinded to the experimental group assignments throughout the analysis process. The codes were broken only after all quantifications were completed. Data from multiple pictures per sample were averaged to represent that sample.

### 2.9 Western Blot

The cell pellets were lysed using radioimmunoprecipitation assay (RIPA) lysis buffer (P0013B, Beyotime) or a nuclear and cytoplasmic protein extraction kit (P0028, Beyotime) supplemented with protease and phosphatase inhibitor cocktail (P1045, Beyotime) according to the manufacturer's instructions. Protein concentrations were determined via a bicinchoninic acid (BCA) protein assay kit (P0010, Beyotime) with bovine serum albumin (BSA) as the standard. Equal amounts of protein (20–30 μg) were separated via 10% sodium dodecyl sulphate‐polyacrylamide gel electrophoresis (SDS‐PAGE) and electrophoretically transferred to polyvinylidene difluoride (PVDF) membranes. The membranes were blocked with 5% nonfat milk (P0216, Beyotime) in Tris‐buffered saline containing 0.1% Tween‐20 (TBST) for 2 h at room temperature. The membranes were then incubated overnight at 4°C with primary antibodies diluted in blocking solution. The primary antibodies used were as follows: rabbit anti‐GAPDH (A19056, ABclonal; 1:5000), mouse anti‐Lamin B1 (66095‐1‐Ig, Proteintech; 1:5000), rabbit anti‐phospho‐AKT (Ser473, 4060S, Cell Signaling Technology [CST]; 1:2000), rabbit anti‐AKT (ab179463, Abcam; 1:2000), rabbit anti‐phospho‐GSK3β (Ser9, ab75814, Abcam; 1:1000), rabbit anti‐GSK3β (22104‐1‐AP, Proteintech; 1:2000) and rabbit anti‐β‐catenin (8480S, CST; 1:1000). Following TBST washes, the membranes were incubated with a horseradish peroxidase (HRP)‐conjugated goat anti‐rabbit secondary antibody (A0208; Beyotime; 1:5000) for 1 h at room temperature. The protein bands were visualised via enhanced chemiluminescence (ECL) substrate and imaged with a Tanon 4600 chemiluminescent imaging system. The original, uncropped western blot images have been provided in Supplementary Data S1. Band intensities were quantified via ImageJ software.

### 2.10 Statistical Analysis

Statistical method selection was guided by data distribution properties. The normality of the data was tested through the Shapiro–Wilk normality test. When normality assumptions were satisfied, parametric approaches were utilised: an unpaired *t*‐test or ANOVA for data meeting homogeneity of variance, supplemented by Tukey's post hoc test. Where variance homogeneity was absent, Welch‐corrected tests (*t*‐tests or ANOVAs) with Dunnett T3 post hoc analysis were applied. For nonnormally distributed data, nonparametric alternatives, specifically the Kruskal–Wallis rank sum test followed by Conover's nonparametric all‐pairs comparison test, were employed. The data are presented as the means ± standard deviations (SDs). Statistical significance was defined as *p* < 0.05, with significance thresholds set at *p < 0.05, **p < 0.01, ***p < 0.001 and ****p < 0.0001. All the statistical analyses described above were conducted via GraphPad Prism version 8.0.1. The BBB scores were analysed using linear mixed‐effects models in R software (version 4.3.1) with the lme4 package to handle missing data and account for repeated measures. The model included treatment group, time point (as a categorical factor), and their interaction as fixed effects, with a random intercept for each animal. Post hoc pairwise comparisons were performed using the emmeans package with Tukey's adjustment.

## 3 Results

### 3.1 Characterisation and Biocompatibility of Multichannel Collagen Scaffolds

**FIGURE 1:**
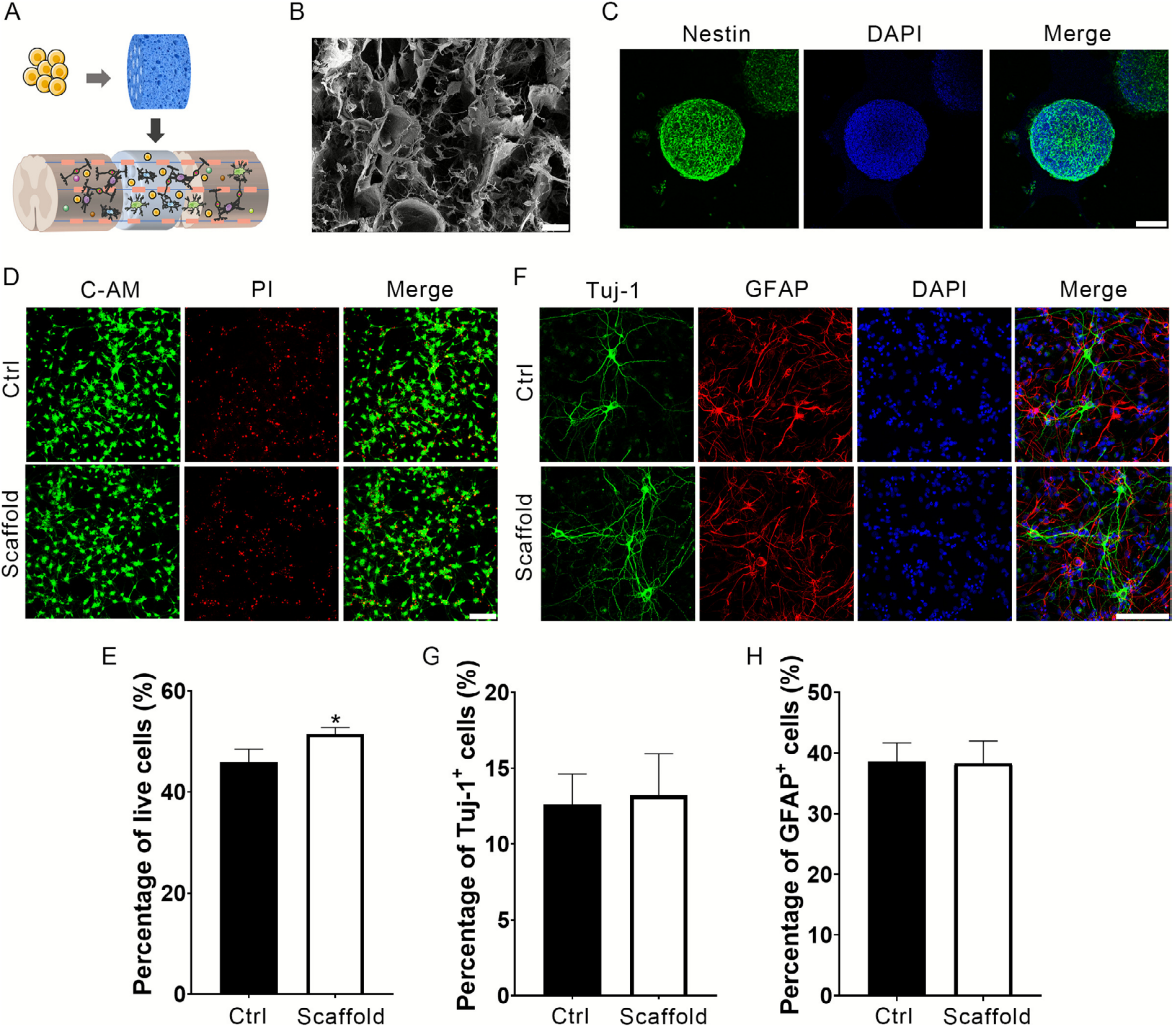
Characterisation and biocompatibility of multichannel collagen scaffolds. (A) Schematic representation of multichannel collagen scaffolds loaded with NSCs. (B) SEM image showing the porous morphology of the collagen scaffold. Scale bars: 100 μm. (C) Neurospheres generated from NSCs positively expressed Nestin. Scale bars: 100 μm. (D) Confocal microscopy images of NSCs cultured for 5 days and stained with C‐AM/PI for double labelling. Live cells were identified as green (C‐AM), while dead cells were stained red (PI). Scale bars: 100 μm. (E) Quantitative analysis of NSC viability demonstrates the survival rate of NSCs in the scaffold. (F) Representative images of immunostaining for neurons (Alexa Fluor 488, green) and astrocytes (Alexa Fluor 568, red). Scale bars: 100 μm. (G, H) Quantification of Tuj‐1‐positive and GFAP‐positive cells. The data represent the means ± SDs (*n* = 3 independent experiments, unpaired *t*‐test).

The multichannel collagen scaffold, which was cylindrical in shape and 3 mm in diameter and thickness, was perforated with axially aligned conduits to load NSCs and promote their directional growth (Figure 1A). The morphological characteristics of the collagen scaffold were identified via SEM. A porous microstructure that enabled the loaded cells to proliferate and differentiate was observed in the scaffold (Figure 1B).

To detect the biocompatibility of the scaffold, primary NSCs from the E14.5 hippocampus were cultured into neurospheres with diameters of 100–200 μm and identified as Nestin‐positive (Figure 1C). Individual NSCs were subsequently adherently cultured and differentiated on the collagen scaffold. Both calcein‐AM/PI live/dead staining results indicated that cell viability slightly increased with the collagen scaffold (Figure 1D,E). In addition, the differentiation capacity of NSCs was detected through immunofluorescence with Tuj‐1/GFAP double staining. NSCs placed on the collagen scaffold were able to differentiate normally (Figure 1F). Statistical analysis revealed almost identical neuronal and astrocytic differentiation to that observed in the control group (Figure 1G,H).

### 3.2 Transplantation of NSC‐Loaded Multichannel Collagen Scaffolds Promotes Functional and Structural Recovery After SCI

**FIGURE 2:**
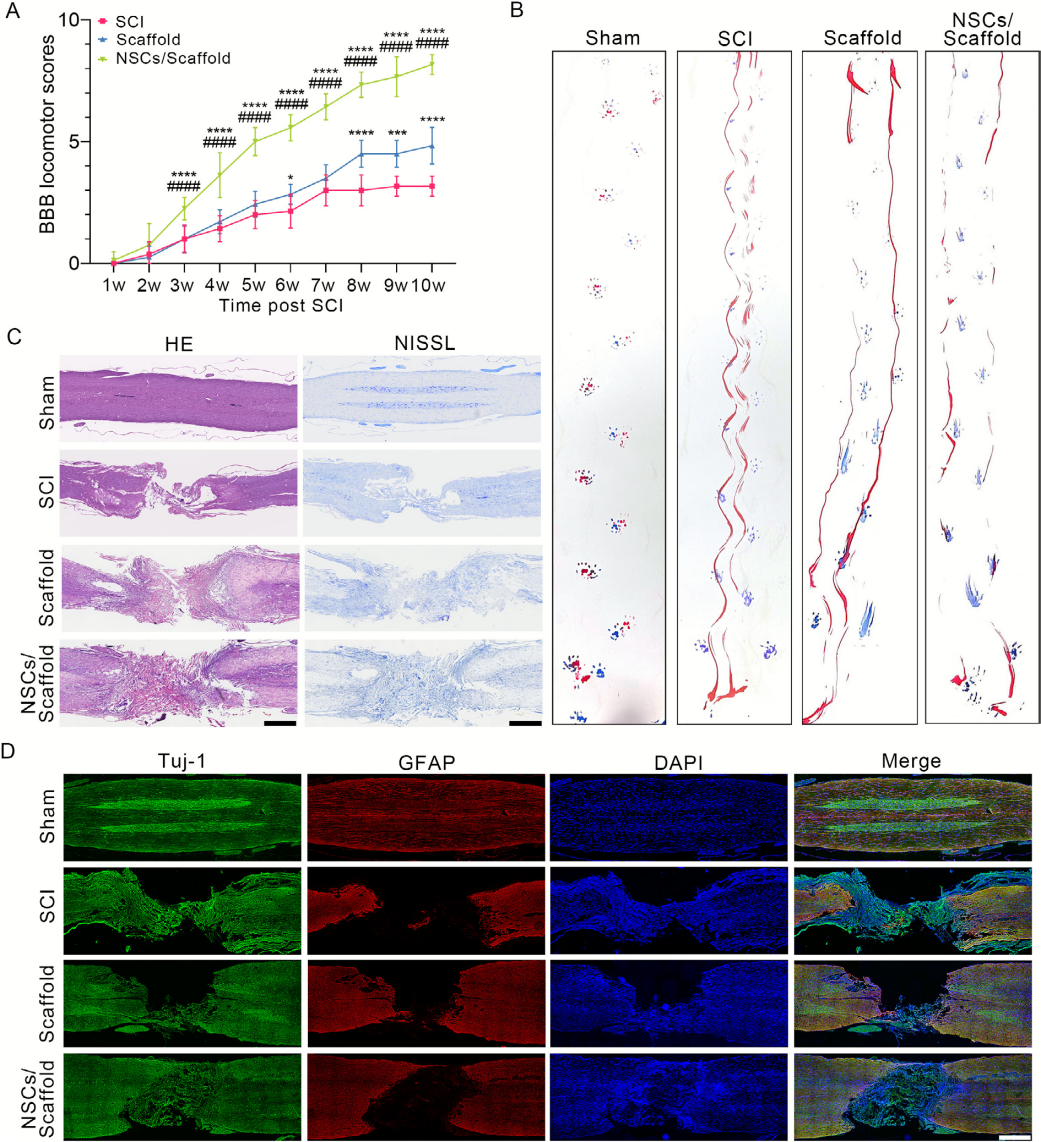
Transplantation of NSC‐loaded multichannel collagen scaffolds promoted functional and structural recovery after SCI. (A) BBB scores were assessed at the indicated time points after SCI. Data are presented as the means ± SDs. Statistical significance was determined by linear mixed‐effects models with Tukey's post hoc test. The sample size for each group at each time point was as follows: SCI group: *n* = 8 at Weeks 1 and 2; *n* = 7 at Weeks 3–6; *n* = 6 at Weeks 7–10. Scaffold group: *n* = 8 at Weeks 1–3; *n* = 7 at Weeks 4 and 5; *n* = 6 at Weeks 6–10. NSCs/scaffold group: *n* = 8 at Weeks 1–4; *n* = 7 at Weeks 5–7; *n* = 6 at Weeks 8–10. *p < 0.05, ***p < 0.001, ****p < 0.0001 compared with the SCI group; ####*p* < 0.0001 compared with the scaffold group. (B) Representative footprint images from rats 10 weeks post‐injury. Blue: Forepaw print; red: Hindpaw print. *n* = 6 per group. (C) Representative panoramic images of spinal cord sections from the indicated groups were analysed by H&E staining and Nissl staining. *n* = 3 per group. Scale bars: 1 mm. (D) Representative immunofluorescence images of longitudinal spinal cord sections 10 weeks post‐SCI stained for Tuj‐1 (green, neurons) and GFAP (red, astrocytes). *n* = 3 per group. Scale bars: 1 mm.

Single NSCs were seeded into the luminal cavities of multichannel collagen scaffolds and implanted into the injured area of the T8 spinal cord. In this study, a total of 24 rats underwent SCI surgery (*n* = 8 for the SCI, scaffold and NSCs/scaffold groups). Six rats underwent a laminectomy. Six animals succumbed to complications secondary to severe autonomic dysreflexia and urinary tract infections during the post‐operative period prior to the end point. The BBB score was used to assess the recovery of motor functions of the hind limb after NSC transplantation in SCI rats. After the spinal cord was transected at the T8 segment, all the rats lost sensory and hind limb movement, with BBB scores of 0 (except those in the sham group, with a BBB score = 21). The rats in the SCI group spontaneously recovered from hindlimb paralysis, with BBB scores ranging from 2 to 3 at Week 10 after surgery. There was only a slight increase in the BBB score in the scaffold transplant group. However, the implantation of NSC‐seeded scaffolds significantly enhanced locomotor recovery. BBB scores were significantly different between the NSC‐treated group and the SCI control group from post‐injury Week 3 through Week 10 (Figure 2A; Table S1). Footprint analysis consistently revealed significant improvements in hindlimb motor function in rats in the NSC transplantation group compared with those in rats in the SCI group or scaffold group (Figure 2B).

H&E and Nissl staining were performed to evaluate histological alterations in the injured spinal cord across the experimental groups. As shown in Figure 2C, compared with the sham group, both the SCI model group and the scaffold‐only control group exhibited disrupted tissue architecture with irregular cavitation at the lesion site. In contrast, NSC‐seeded scaffold transplantation markedly attenuated tissue damage, as evidenced by enhanced structural reorganisation and increased density of Nissl bodies within the injury zone.

Immunostaining was performed on longitudinal cord sections. Tuj‐1, a neuron‐specific βIII‐tubulin marker, serves as an established early neuronal biomarker [26]. Compared with those in both the scaffold‐only and SCI control groups, the number of Tuj‐1^+^ cells in the NSC‐seeded scaffold group was significantly greater (Figure 2D). In addition, GFAP, a well‐characterised indicator of astrocyte activation in response to CNS injury or stress [27], demonstrated elevated immunoreactivity at lesion sites in the scaffold‐only and SCI groups. In contrast, the NSC‐seeded scaffold group exhibited attenuated reactive astrogliosis (Figure 2D). These findings align with our prior studies, demonstrating that NSC‐loaded multichannel collagen scaffolds significantly enhance neural restoration in rats with complete SCI‐induced paraplegia.

### 3.3 Metabolomic Profiling Reveals NSC‐Mediated Metabolic Reprogramming

To elucidate the metabolic mechanisms underlying NSC transplantation therapy for SCI, untargeted metabolomic analysis was performed on spinal cord samples from four experimental groups: sham (*n* = 9), SCI (*n* = 10), scaffold‐only (*n* = 8) and NSCs/scaffold (*n* = 9). Raw data processing identified 5974 metabolic features (positive mode: 3522 and negative mode: 2272). The robustness of the analytical platform was evaluated via the use of QC samples. The coefficient of variation (CV) for all detected metabolites in the QCs was less than 20%. The list of reference matched metabolites with CV values is provided in the supplemental material (Table S2). This high reproducibility was further confirmed by the tight clustering of the QC samples in the principal component analysis (PCA) score plot (Figure S1), indicating minimal technical variation throughout the data acquisition process.

**FIGURE 3:**
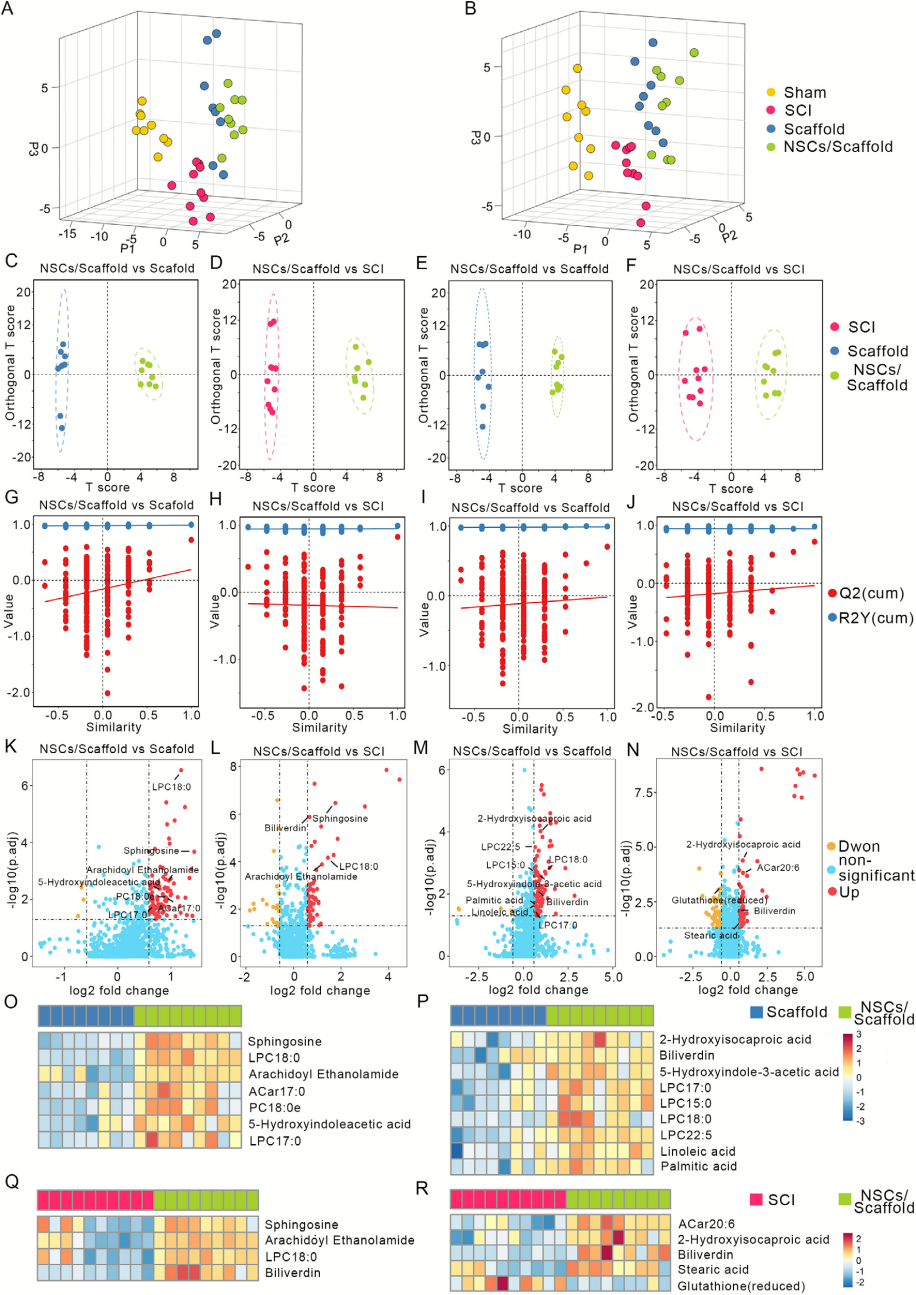
Metabolomic profiling reveals NSC‐mediated metabolic reprogramming in the injured spinal cord. Three‐dimensional score plots from PLS‐DA analysis of metabolomic profiles acquired in (A) positive and (B) negative ionisation modes. OPLS‐DA score plots comparing the NSCs/scaffold group vs. the scaffold group in panels (C) (positive mode) and (E) (negative mode), and vs. the SCI group in panels (D) (positive mode) and (F) (negative mode). Permutation test validation plots (200 permutations) of the OPLS‐DA models comparing NSCs/scaffold vs. scaffold in panels (G) (positive mode) and (I) (negative mode), and NSCs/scaffold vs. SCI in panels (H) (positive mode) and (J) (negative mode). Volcano plots comparing metabolite profiles between NSCs/scaffolds and scaffolds in panels (K) (positive mode) and (M) (negative mode), and between NSCs/scaffolds and SCIs in panels (L) (positive mode) and (N) (negative mode). Heatmaps illustrating the relative abundance of differentially abundant metabolites identified between the (O, Q) NSCs/scaffold and scaffold groups (O, positive mode; Q, negative mode) and (P, R) NSCs/scaffold and SCI groups (P, positive mode; R, negative mode). Lipid metabolites are abbreviated as “C *x*:*y*,” where *x* = total carbon atoms and *y* = total double bonds in fatty acyl chains.

Initial PLS‐DA visualisation revealed pronounced metabolic separation between the sham‐operated and injury groups, with discernible clustering trends among the SCI, scaffold‐only and NSCs/scaffold groups in both ionisation modes (Figure 3A,B). Although PLS‐DA achieves high classification accuracy, its usefulness is restricted because the numerous analytes affect model performance. To increase model specificity for biomarker discovery, OPLS‐DA was employed for pairwise comparisons. OPLS‐DA score plots revealed distinct metabolic profiles between the NSCs/scaffold group and either the scaffold‐only group or the SCI control group (Figure 3C–F). Model validation parameters confirmed robustness (positive mode: NSCs/scaffold vs. scaffold (*R* ^2^ *Y* = 0.992, *Q* ^2^ = 0.720); NSCs/scaffold vs. SCI (*R* ^2^ *Y* = 0.992, *Q* ^2^ = 0.826); negative mode: NSCs/scaffold vs. scaffold (*R* ^2^ *X* = 0.994, *Q* ^2^ = 0.703); NSCs/scaffold vs. SCI (*R* ^2^ *Y* = 0.976, *Q* ^2^ = 0.715)). All the models met validity criteria (*R* ^2^ *Y*/*Q* ^2^ > 0.5). Permutation testing (200 iterations) and CV‐ANOVA (*p* < 0.05 for all models) confirmed the absence of overfitting, as evidenced by permuted *R* ^2^/*Q* ^2^ values lower than the original *R* ^2^/*Q* ^2^ values and negative *Q* ^2^ regression intercepts (Figure 3G–J). With CV‐ANOVA *p* values less than 0.05 for each OPLS‐DA model, all models were dependent.

The volcano plots revealed the differences in the metabolic profiles among the SCI, scaffold and NSCs/scaffold groups corresponding to each OPLS‐DA score plot (Figure 3K–N). Differentially abundant metabolites were selected on the basis of the OPLS‐DA model (VIP ≥ 1), fold change (|FC| ≥ 1.5) and adjusted *p* < 0.05. We identified 15 metabolites distinguishing NSCs/scaffolds from scaffold‐only groups and 9 differentiating NSCs/scaffolds from SCI. Hierarchical clustering of these metabolites revealed distinct abundance patterns across groups (Figure 3O–R), encompassing neurotransmitters (5‐hydroxyindoleacetic acid), fatty acids and derivatives (palmitic acid, stearic acid, linoleic acid, Acar17:0), sphingolipids (sphingosine), lysophosphatidylcholines (LPC15:0, LPC17:0, LPC18:0 and LPC22:5), endocannabinoids (arachidonoyl ethanolamine) and others. These findings demonstrate that NSC transplantation significantly remodels the metabolic microenvironment post‐SCI.

### 3.4 LPC18:0 Promotes Functional and Structural Recovery After SCI Rather Than Sphingosine

Metabolic analysis revealed that sphingosine and LPC18:0 were the two most elevated metabolites identified in the NSCs/scaffold group compared with the scaffold‐only group. Furthermore, the concentrations of these metabolites were markedly greater in the NSC‐treated rats than in the untreated controls. Consequently, sphingosine and LPC18:0 were selected as candidate bioactive metabolites. Following metabolomic studies, the identification and validation of these metabolites of interest are essential for subsequent steps. High‐purity standards of LPC18:0 and sphingosine were analysed under identical UHPLC‐Q/TOF‐MS conditions as those employed in the metabolomic study. The UHPLC retention times (data not shown) and MS/MS spectra were in close agreement between the tissue samples and authenticated standards (Figure S2).

**FIGURE 4:**
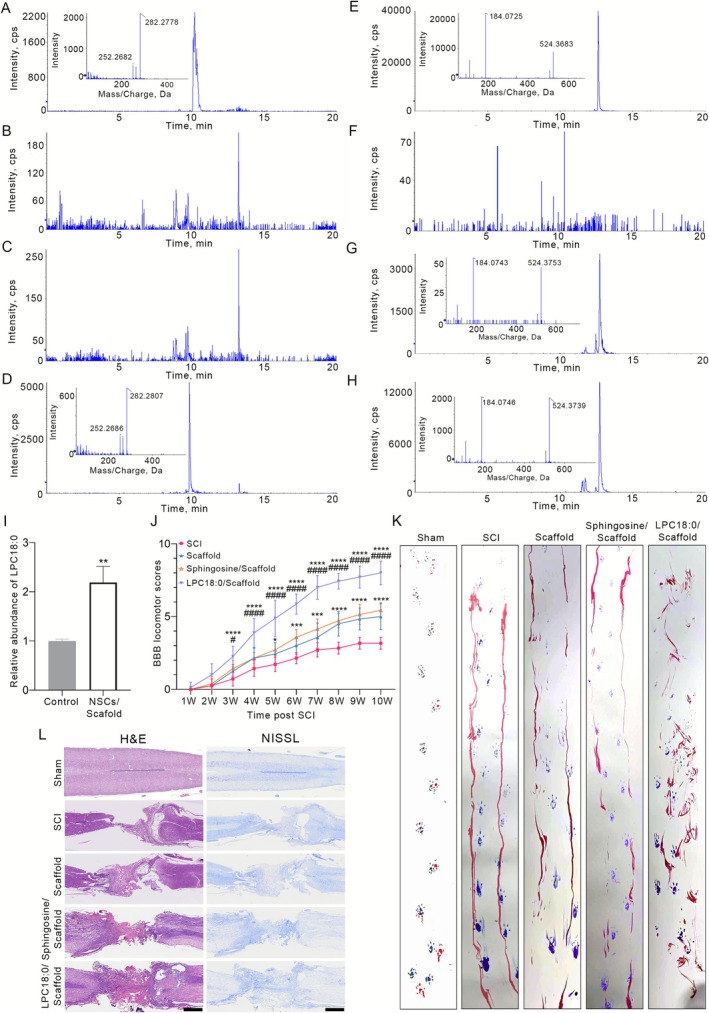
Effects of sphingosine or LPC18:0 administration on SCI rats. (A–D) Extracted ion chromatograms for sphingosine (*m/z* 300.29) in (A) authentic standard medium, (B) basic DMEM/F12 medium, (C) NSC culture supernatant and (D) NSC cell extract. The insets show the corresponding MS/MS spectra. (E–H) Extracted ion chromatograms for LPC18:0 (*m/z* 524.37) in (E) authentic standard solution, (F) basic DMEM/F12 medium, (G) NSC culture supernatant and (H) NSC cell extract. The insets show the corresponding MS/MS spectra. (I) Relative abundance of LPC18:0 in the supernatant of NSCs cultured conventionally or cocultured with a collagen scaffold normalised to the internal standard (LPC12:0). The data represent the means ± SDs (*n* = 3 independent cultures; **p < 0.01, unpaired *t*‐test). (J) BBB scores were assessed at the indicated time points after SCI with/without sphingosine or LPC18:0. The data represent the means ± SDs. Statistical significance was determined by linear mixed‐effects models with Tukey's post hoc test. The sample size for each group at each time point was as follows: SCI group: *n* = 8 at Weeks 1 and 2; *n* = 7 at Weeks 3–7; *n* = 6 at Weeks 8–10. Scaffold group: *n* = 8 at Weeks 1–3; *n* = 7 at Weeks 4–7; *n* = 6 at Weeks 8–10. Sphingosine/scaffold group: *n* = 8 at Weeks 1 and 2; *n* = 7 at Weeks 3–10. LPC18:0/Scaffold group: *n* = 8 at Weeks 1–5; *n* = 7 at Weeks 5–10. *p < 0.05, ***p < 0.001 and ****p < 0.0001 compared with the SCI group; #*p* < 0.05 and ####*p* < 0.0001 compared with the scaffold group. (K) Representative footprint images from rats 10 weeks post‐injury. Blue: Forepaw print; red: Hindpaw print. *n* = 7 per group. (L) Representative images of H&E and Nissl staining of spinal cord sections from the indicated groups. *n* = 3 per group. Scale bars: 1 mm.

Following the identification and characterisation of sphingosine and LPC18:0 as described, we investigated whether these metabolites originate directly from NSCs. Sphingosine and LPC18:0 were detected in the cell extracts and culture supernatants using UHPLC‐Q/TOF‐MS. Sphingosine was exclusively detected in the cell extract, with no measurable levels observed in the corresponding supernatant or in the basic DMEM/F12 medium alone (Figure 4A–D). In contrast, LPC18:0 was detected in both the cell extracts and the supernatants but was not detected in the basic DMEM/F12 medium (Figure 4E–H). To investigate the potential influences of the collagen scaffold, we compared LPC18:0 levels in supernatants derived from conventionally cultured NSCs with those in supernatants cocultured with the collagen scaffold. The relative concentrations of LPC18:0 were determined via the use of LPC12:0 as an internal standard and were calculated via normalisation of the metabolite peak areas to those of the internal standard. Notably, the LPC18:0 concentration in supernatants from NSCs cocultured with the scaffold was approximately twofold greater than that in supernatants from control NSCs (Figure 4I). We speculated that collagen‐based materials may increase the metabolic activity of NSCs, potentially promoting increased secretion of LPC18:0.

To further evaluate the therapeutic potential of LPC18:0 and sphingosine in injured spinal cord repair, we assessed functional recovery following their administration in an SCI model. In this study, a total of 32 rats underwent SCI surgery (*n* = 8 for the SCI, scaffold, sphingosine/scaffold and LPC18:0/scaffold groups) and 6 rats underwent laminectomy. Six animals succumbed to complications secondary to severe autonomic dysreflexia and urinary tract infections during the post‐operative period prior to the end point. According to the BBB locomotor rating scale, in situ administration of 10 μM LPC18:0, but not sphingosine, significantly enhanced locomotor recovery post‐SCI (Figure 4J; Table S1). Footprint analysis further confirmed improved hindlimb motor function in the LPC18:0‐treated group, which was consistent with the BBB scores (Figure 4K). Histopathological evaluation via H&E and Nissl staining of injured spinal cord tissues from the five experimental groups revealed that LPC18:0 treatment markedly reduced the lesion area. Concomitantly, only the LPC18:0 group exhibited a significant increase in Nissl‐positive neuronal density at the injury site (Figure 4L). These data demonstrate that 10 μM LPC18:0 promotes both functional and structural repair after SCI. Although the 10 μM dose of LPC18:0 was validated rationally through both in vitro cytotoxicity assays and in vivo gradient administration (Figure S3), a limitation of this study is that we did not directly quantify the retention and concentration time course of LPC18:0 in situ, which we plan to address in future work via advanced analytical techniques.

### 3.5 NSC‐Derived LPC18:0 Enhances Nerve Regeneration in SCI Rats

**FIGURE 5:**
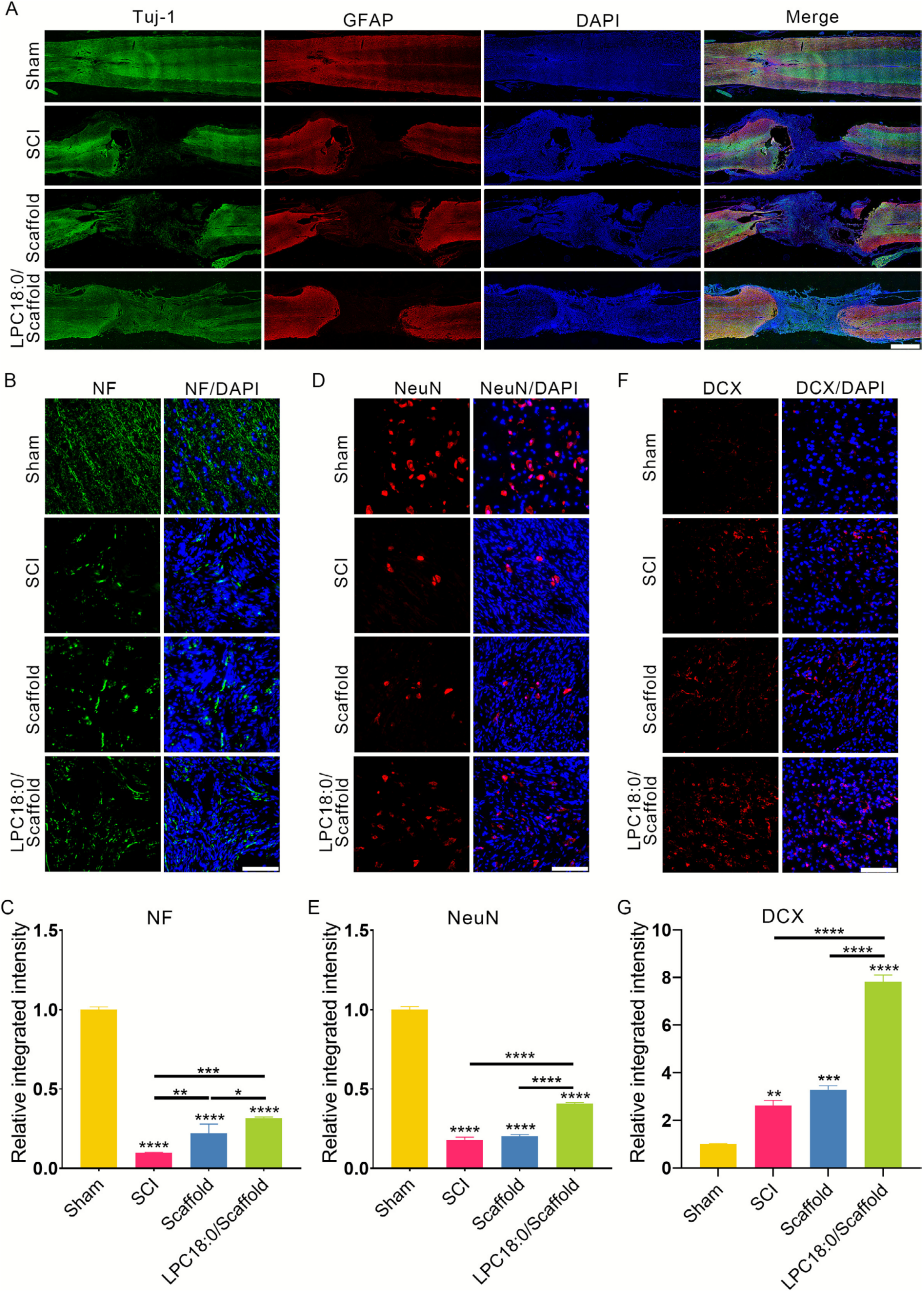
LPC18:0 administration promotes neuronal regeneration in SCI rats. (A) Representative immunofluorescence images of longitudinal spinal cord sections 10 weeks post‐SCI stained for Tuj‐1 (green, neurons) and GFAP (red, astrocytes). Scale bar: 1 mm. Representative high‐magnification immunofluorescence images (400×) at the lesion epicentre showing (B) neurofilament (NF, green), (D) neuronal nuclei (NeuN, red) and (F) doublecortin (DCX, red) staining. Nuclei were counterstained with DAPI (blue). Scale bars: 100 μm. (C, E, G) Quantitative analysis of (C) NF^+^ axonal density (integrated intensity), (E) NeuN^+^ mature neuronal density and (G) DCX^+^ immature neuronal density at the lesion site. The data represent the means ± SDs (*n* = 3 rats/group). *p < 0.05, **p < 0.01, ***p < 0.001 and ****p < 0.0001 (one‐way ANOVA with Tukey's post hoc test).

Neural circuit reconstruction underlies motor functional recovery following SCI, and consequently, immunofluorescence assays employing multiple antibodies have been conducted to evaluate neural regeneration. The tissue sections were costained with Tuj‐1 (neuronal marker) and GFAP (astrocytic marker). As shown in Figure 5A, LPC18:0 administration significantly increased Tuj‐1^+^ cell density at the lesion site compared with that in the scaffold‐only and SCI control groups, whereas GFAP^+^‐reactive hypertrophic astrocyte levels remained comparable between the LPC/scaffold and scaffold groups. NeuN, a canonical mature neuronal marker, revealed significantly greater NeuN^+^ neuronal density in the injury epicentre following LPC18:0 treatment than in the SCI and scaffold control groups (Figure 5B,C). One notable characteristic of SCI is the lack of axonal regeneration, which causes the disintegration of neural circuits and lasting neurological issues [28]. Thus, spinal cord sections were labelled with a neurofilament antibody to assess axon regeneration. Ten weeks after surgery, NF immunoreactivity was mainly observed throughout the white matter of the spinal cord. Quantification revealed enhanced axonal regeneration in LPC18:0‐treated animals compared to untreated control animals (Figure 5D,E). These findings indicate that local LPC18:0 administration substantially promotes neuroprotection and axonal repair.

Given that the neurons are incapable of proliferating, we suspected that the newborn neurons predominantly arose from the differentiation of endogenous NSCs. Sections from 7 days post‐injury were immunostained with DCX (a marker for immature neurons and neuronal precursors) [29]. Consistent with previous reports, DCX^+^ cells were rarely detected in the tissue of sham‐operated rats but could be observed after SCI surgery. Most importantly, LPC18:0 treatment significantly increased DCX^+^ cell counts relative to those in all the other groups (Figure 5F,G). Collectively, these data demonstrate that LPC18:0 potently enhances injury‐site neurogenesis, contributing to functional recovery after SCI.

### 3.6 LPC18:0 Promotes Neural Differentiation via the GPR55–AKT–GSK3β Pathway

**FIGURE 6:**
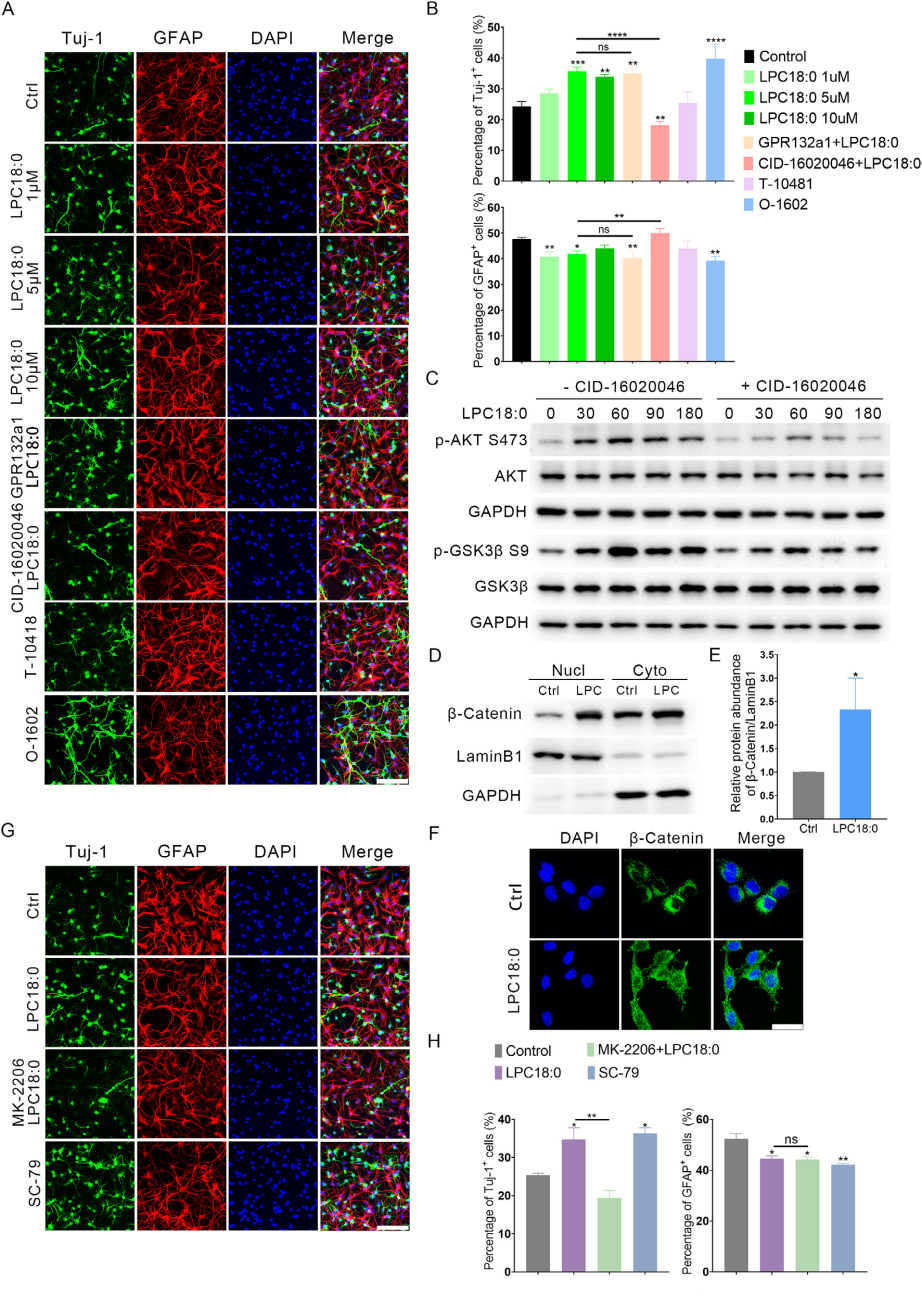
LPC18:0 promotes neural differentiation via the GPR55–AKT–GSK3β pathway. (A, G) Representative immunofluorescence images of NSCs differentiated under the indicated conditions: (A) Vehicle control (Ctrl), LPC18:0 (1, 5 and 10 μM), GPR132 antagonist 1 (GPR132a1, 5 μM), CID16020046 (GPR55 antagonist, 5 μM), T‐10418 (GPR132 agonist, 5 μM), O‐1602 (GPR55 agonist, 5 μM); (G) Vehicle control (Ctrl), LPC18:0 (5 μM), LPC18:0 + MK‐2206 (AKT inhibitor, 1 μM) and SC‐79 (AKT activator, 5 μM). The cells were stained for Tuj‐1 (neurons, green) and GFAP (astrocytes, red). Nuclei were counterstained with DAPI (blue). Scale bars = 100 μm. (B, H) Quantification of Tuj‐1^+^ neurons and GFAP^+^ astrocytes for the conditions shown in (B) panel A and (H) panel G. (*n* = 3 independent experiments). (C) Western blot analysis of phosphorylated AKT (Ser473) and phosphorylated GSK3β (Ser9) levels in NSCs treated with LPC18:0 (5 μM) with or without pretreatment with CID16020046 (5 μM). (D) Western blot analysis of β‐catenin levels in cytoplasmic (cyto) and nuclear (nucl) fractions of NSCs treated with LPC18:0 (5 μM) or vehicle control (Ctrl). Lamin B1 and GAPDH were used as loading controls for nuclear and cytoplasmic fractions, respectively. (E) Densitometric quantification of β‐catenin protein levels normalised to Lamin B1 (nuclear) or GAPDH (cytoplasmic) from three independent experiments (*n* = 3). (F) Representative immunofluorescence images showing β‐catenin (green) localisation in NSCs treated with LPC18:0 or vehicle control. Nuclei were counterstained with DAPI (blue). Scale bars: 20 μm. The data represent the means ± SDs. Statistical analysis was performed via one‐way ANOVA with Tukey's post hoc test (B, H) or an unpaired *t*‐test (E). *p* values are denoted as follows: *p < 0.05, **p < 0.01, ***p < 0.001 and ****p < 0.0001.

In vitro differentiation assays demonstrated that LPC18:0 promoted neuronal differentiation in a concentration‐dependent manner (Figure 6A,B). Protein and metabolite interactions are vital for various cellular and physiological activities within biological systems. LPC18:0 was found to potentially interact with the peroxisome proliferator‐activated receptor gamma (PPARγ) [30]. Thus, we evaluated the effects of LPC18:0 on the transcriptional activity of PPARγ in HEK‐293T cells via a PPAR response element (PPRE)‐luciferase assay. LPC18:0 did not increase PPRE luciferase activity, suggesting that PPARγ‐independent signals mediate the effect of LPC18:0 on NSC differentiation (Figure S4). Previous investigations revealed that GPCRs, such as GPR132 and GPR55, serve as effectors of LPCs [31, 32]. To explore the roles of these receptors in LPC18:0‐induced neurogenesis, NSCs were pretreated with either GPR132 antagonist 1 or CID16020046 (an antagonist of GPR55) prior to LPC18:0 exposure. The results indicated that LPC18:0‐induced neuronal differentiation was mediated by GPR55, as evidenced by inhibition with CID16020046, but not by GPR132 antagonism (Figure 6A,B). Consistent with previous results [33], pharmacological activation studies confirmed that the GPR55 agonist O‐1602 increased neuronal differentiation, whereas the GPR132 agonist T10418 had no significant effect (Figure 6A,B). Given the established role of the AKT signalling pathway in regulating cellular processes, including proliferation and differentiation within neural systems, we examined its involvement. Western blot analysis revealed that LPC18:0 treatment rapidly increased the phosphorylation of AKT and its downstream substrate GSK3β in NSCs. As expected, this activation was abrogated by pretreatment with the GPR55 antagonist CID16020046 (Figure 6C). Phosphorylation of Ser9 inhibits GSK3β activity, thereby disrupting the function of the β‐catenin degradation complex and inducing the nuclear translocation of β‐catenin. Given the established role of AKT/GSK3β signalling in regulating β‐catenin stability, we next examined the subcellular localisation of β‐catenin. We found that LPC18:0 treatment significantly increased β‐catenin levels in both the cytoplasmic and nuclear fractions (Figure 6D,E) and immunocytochemistry confirmed its nuclear accumulation (Figure 6F). Finally, we assessed the involvement of the AKT pathway in NSC differentiation. SC‐79 (an activator of AKT) treatment significantly increased the percentage of Tuj‐1‐positive cells compared with that in the control groups, whereas blocking the AKT signalling pathway with an AKT antagonist (MK‐2206) markedly impaired neurogenesis (Figure 6G,H). These results indicate that LPC18:0 promotes neural differentiation via the GPR55–AKT–GSK3β pathway.

## 4 Discussion

Despite decades of research, SCI remains one of the most devastating neurological conditions for which no curative therapy exists. Currently, the advancements of clinical cell‐based therapies offer transformative potential for conditions previously considered untreatable. A range of candidate cell types, such as NSCs, mesenchymal stem cells and induced pluripotent stem cells, have been investigated for their curative effects on SCI [34, 35, 36]. Among these, NSCs have garnered significant interest because of their innate neurogenic differentiation capacity and ability to secrete neurotrophic molecules. The therapeutic mechanisms of NSCs include neuroprotection, immunomodulation, myelin regeneration and axonal outgrowth. For exogenous NSC transplantation, biomaterial scaffolds are increasingly employed to provide structural support and increase graft survival. In our previous work and the current work [23], we engineered an implantable porous collagen scaffold with axially aligned microchannels optimised for NSC delivery at SCI lesion sites. This construct exhibited favourable biocompatibility and supported NSC viability, proliferation and differentiation. Subsequent in vivo validation confirmed that NSC‐loaded scaffold transplantation into complete transection SCI models contributed to significant functional recovery, as evidenced by improved locomotor performance.

Substantial preclinical and clinical evidence support the neuroregenerative potential of NSCs in SCI. Consistent with these reports, we observed an increased density of Tuj‐1^+^ neurons and Nissl bodies at the lesion site in NSCs/scaffold‐treated rats relative to untreated controls. Previous studies have demonstrated that grafted NSCs can differentiate into neurons that integrate into the host neural circuitry, potentially replacing damaged tissue [37, 38]. However, secondary pathological processes such as neuroinflammation, excitotoxicity and oxidative stress occur, contributing to metabolic disorders in and around the injury area. Emerging evidence indicates that the dysregulated SCI microenvironment compromises the long‐term survival and neuronal differentiation of transplanted NSCs [5]. The fate of transplanted NSCs predominantly leans towards astrocytes, with restricted neurogenesis and oligogenesis [39]. Given the limited neuronal differentiation of transplanted NSCs, we suspect that newborn neurons in the NSCs/scaffold cotransplanted animals are predominantly derived from differentiated endogenous NSCs rather than from exogenous NSCs themselves [40]. Consistent with this perspective, our previous study revealed that the improved motor function after SCI due to allogeneic NSC transplantation is largely attributed to the secretion of neurotrophic factors rather than to the direct replacement of neurons by those differentiated from the transplanted cells [41]. Multiple neurotrophic factors secreted by NSCs, such as VEGF, BDNF, GDNF, EGF, IGF‐1/2 and NGF, play a role in their neuroregenerative effects on SCI. For example, GDNF‐overexpressing NSCs dramatically enhanced neuronal differentiation in both in vitro and in vivo models [42]. Recent evidence further revealed that small molecules such as nicotinamide riboside can promote SCI recovery by acting on endogenous NSCs [43]. These findings motivated our investigation into whether transplanted NSCs enhance neural repair via metabolic microenvironment remodelling at the lesion site.

With the rapid evolution of high‐throughput analytical techniques, metabolomics has increasingly been applied to research related to SCI. Studies have demonstrated a range of metabolic changes in spinal cord tissue, cerebrospinal fluid and serum after SCI, which can be used as biomarkers to reflect the severity of the injury and to monitor its progression and treatment response in animal studies [16, 44]. Moreover, promising outcomes have been observed with metabolic reprogramming therapy in cellular and animal models of SCI, leading to the restoration of function and regeneration of neural axons [45]. However, the metabolic reprogramming mediated by exogenous NSCs in SCI treatment remains essentially unstudied. In the present research, UHPLC‐Q/TOF‐MS‐based metabolomic analysis was first applied to characterise the metabolic signatures of the spinal cord in SCI rats with or without NSC transplantation, as well as in sham‐operated rats. Data analysis via the OPLS‐DA model subsequently allowed for the screening of differentially abundant metabolites. Comparative analysis revealed significant differences in the levels of endogenous metabolite classes (fatty acids, amino acids, lipids and bile acids) between the sham and SCI groups. The levels of proinflammatory mediators, such as 5‐hydroxyindoleacetic acid and asymmetric dimethylarginine, were elevated, whereas the levels of anti‐inflammatory mediators, such as nicotinamide and glutathione, were decreased post‐SCI. An imbalance in energy metabolism and lipid metabolism was also observed after trauma. These metabolic alterations may exacerbate secondary pathogenesis, promoting neuronal death, scar formation and neuroinflammation, ultimately contributing to poor neurological outcomes. To delineate NSC‐mediated metabolic modulation, we compared metabolite profiles across the NSCs/scaffold, scaffold‐only and SCI groups. Metabolomic analysis revealed sphingosine and LPC18:0 as the most significantly upregulated metabolites in the NSC‐treated group relative to the SCI control group. However, no statistically significant increase in these compounds was detected in the scaffold‐only implantation group, indicating that their increase was specifically mediated by NSC transplantation rather than the biomaterial carrier. Furthermore, both metabolites were detected in the NSC supernatant and/or cellular extract. Taken together, sphingosine and LPC18:0 were prioritised for subsequent investigations to define NSC‐driven metabolic regulation in the post‐SCI microenvironment.

The functional significance of sphingosine and LPC18:0 in SCI remains incompletely characterised. Evaluating individual metabolites with functional significance would assist in the transition to clinical development. We therefore investigated their functional roles in our SCI model. Although a previous study indicated that chronic application of sphingosine to spinal cord‐lesioned rats promoted axon sprouting and enhanced functional recovery after SCI [46], our study demonstrated no significant therapeutic effects of sphingosine on functional recovery following SCI, possibly because of the interconversion between sphingosine and sphingosine 1‐phosphate (S1P), which maintains their physiological concentration at normal levels. Moreover, S1P is considered a proinflammatory agent that can cause an inflammatory response and induce neuronal damage in SCI [47].

Notably, compared with untreated control rats, LPC18:0‐treated SCI rats exhibited significantly enhanced motor functional recovery. Our metabolomic studies revealed that the LPC18:0 level was more than twofold higher in the NSC transplantation group than in the SCI and scaffold‐only groups. Strikingly, compared with sham control rats, SCI rats presented significantly elevated levels of multiple LPC species. Resident cell types in the spinal cord might secrete LPCs under injury conditions, likely because of the upregulation of phospholipase A_2_ (PLA_2_) activity, which generates LPC species through the hydrolysis of the sn‐2 ester bond in glycerophospholipids [48]. These autocrine LPCs may also contribute positively to neuroprotection and regeneration after SCI. However, concentrations of autocrine LPC18:0 may be insufficient to maintain the minimum effective concentration, hence failing to promote functional recovery after SCI.

However, the role of LPCs in the nervous system remains controversial. Some studies have inferred that LPCs are neuroprotective and regenerative. For example, Karaki et al. reported that LPC16:0, LPC18:0 and LPC18:1 might exert anti‐dementia effects by inhibiting α‐Syn aggregation [49]. Similarly, Wuhanqimuge et al. reported that LPCs significantly enhanced the NGF‐induced phosphorylation of MAPK, which could accelerate the neurogenesis of PC12 cells [50]. On the other hand, deleterious effects of LPCs, such as demyelination and pain hypersensitivity, have been observed [51]. This dichotomy underscores that LPC bioactivity is critically dependent on the acyl chain structure, concentration and pathological context. Therefore, in‐depth research focusing on a single type of LPC is warranted.

To elucidate the therapeutic mechanisms of LPC18:0 in SCI, we performed immunofluorescence analysis of neural cells at the lesion epicentre. Our findings demonstrate that LPC18:0 significantly enhances axon regeneration and promotes neurogenesis at the injury site. Furthermore, in vitro analyses confirmed its concentration‐dependent stimulation of neuronal differentiation. Within the spinal cord, endogenous NSCs reside in the central canal ependyma, where they retain their multipotent differentiation capacity across neural lineages [52]. Although they are typically maintained in a quiescent state, these resident NSCs become activated within the SCI environment through inflammatory mediators, reactive oxygen species and glutamate signalling [7]. Most of the native NSCs in the injured spinal cord differentiate into astrocytes, contributing to the glial scar, with only a few developing into oligodendrocytes and neurons [53]. Consequently, the endogenous NSC population has a limited capacity for neuronal replacement and neural circuit restoration following SCI. However, our data suggest that LPC18:0 represents an innovative therapeutic strategy primarily by enhancing endogenous NSC neuronal differentiation, thereby facilitating recovery from SCI.

Building upon our in vivo findings, we subsequently investigated the unresolved question of which receptor serves as the upstream mediator of LPC18:0‐induced neurogenesis. GPR132 and GPR55 represent the most extensively characterised receptors for LPC18:0 [31, 54]. Our data demonstrated that pharmacological inhibition of GPR55, but not GPR132, abolished the pro‐neurogenic effects of LPC18:0. Moreover, activating GPR55 with O‐1602 (a GPR55 activator) significantly increased the neuronal differentiation ratio in NSC cultures, which is consistent with previous research [55]. GPR55 is expressed not only in NSCs but also physiologically in neurons, oligodendrocytes and microglia. For example, activation of GPR55 has been shown to influence neuronal excitability [56]. Therefore, the potential contributions of LPC18:0 to other GPR55‐expressing cell types cannot be excluded and warrant further investigation. In future research, we will conduct a more comprehensive investigation into the role of GPR55 across other cell types. We additionally delineated the downstream signalling pathway. LPC18:0 rapidly induced the phosphorylation of AKT, which increased neuronal differentiation. Subsequently, GSK3β, a major AKT substrate, underwent phosphorylation at serine 9, resulting in its inactivation. This finding aligns with the established literature demonstrating that GSK3β inhibition promotes neurogenesis [57]. Following GSK3β inactivation, β‐catenin dissociates from the degradation complex and translocates to the nucleus. Within the nuclear compartment, β‐catenin forms transcriptional complexes with TCF/LEF factors to upregulate neurogenic genes, including *BDNF*, *CyclinD1* and *NeuroD1*, thereby facilitating NSC differentiation [58].

In conclusion, we systematically characterised metabolic microenvironment alterations at SCI lesion sites following NSC transplantation via metabolomics approaches. Our findings revealed that engrafted NSCs significantly remodel the metabolic landscape of injured spinal tissues. Among the regulated metabolites, LPC18:0 was identified as a potential bioactive mediator. Mechanistically, LPC18:0 promotes endogenous NSC neurogenesis through the GPR55/AKT/GSK3β signalling axis, thereby facilitating functional recovery after SCI. This study elucidates a novel metabolic mechanism underlying NSC‐based therapies for spinal cord repair.

## Author Contributions

D.C. and S.L. were responsible for methodology development, major experimental execution, data analysis and manuscript drafting. L.‐Y.T., M.‐M.Y. and C.‐W.X. were involved primarily in demanding in vivo studies: animal surgery, post‐operative care, behavioural assessments and tissue collection and processing. Y.J. and H.Y. were involved in the metabolomics workflow and bioinformatics analysis. C.‐X.T. and Y.‐N.W. focused on NSC culture and differentiation assays. Y.‐Y.X. was primarily assigned to resource provision and project administration support. P.‐P.S. and B.W. were responsible for the overarching conceptualisation, project direction and administration, major funding acquisition, final validation of findings and extensive manuscript review and editing.

## Ethics Statement

All experiments involving rats were approved by the Ethics Committee of the Affiliated Drum Tower Hospital of Nanjing University Medical School (Project title: Research on the Metabolomic Mechanism of NSC Transplantation in Treating Spinal Cord Injury; approval no. DWSY‐22110898; date of approval: 8 November 2022).

## Conflicts of Interest

The authors declare no conflicts of interest.

## Supporting information


**Figure S1:** Three‐dimensional principal component analysis (PCA) score plot of spinal cord metabolomes from sham (*n* = 9), SCI (*n* = 10), scaffold‐only (*n* = 8) and NSCs/scaffold (*n* = 9) groups. The metabolomic data acquired by LC–MS. Left: positive ion mode; Right: negative ion mode.
**Figure S2:** Identification of Sphingosine and LPC18:0 (A) Representative MS/MS spectra of sphingosine (precursor ion *m/z* 300.29) acquired from standard (top) or tissue samples (bottom). Characteristic fragment ions at *m/z* 252.26, 264.26 and 282.27 were observed. (B) Representative MS/MS spectra of LPC18:0 (precursor ion *m/z* 524.38) acquired from standard (top) or tissue samples (bottom). A characteristic fragment ion at *m/z* 184.07 was observed.
**Figure S3:** (A) Concentration‐dependent cytotoxicity of LPC18:0 on neural stem cells after 24 h of treatment (*n* = 3 independent experiments). Statistical significance was determined by one‐way ANOVA with Tukey's post hoc test compared to the control group. In vitro viability began to decrease at 20 μM. (B) The range of in vivo doses (0–30 μM) was chosen to establish a complete dose–response relationship. This dosing regimen was confirmed to be well‐tolerated in our SCI model. The in vivo doses (0–30 μM) were selected to establish a complete dose–response relationship. All doses were well‐tolerated in the animals, with no signs of systemic toxicity observed throughout the study. The BBB scores were assessed at 10 weeks after SCI following LPC 18:0 treatment at the indicated doses (*n* = 6 per group). Data represent mean ± SD. Statistical significance was determined by one‐way ANOVA with Tukey's post hoc test. *p < 0.05, **p < 0.01, ***p < 0.001 and ****p < 0.0001.
**Figure S4:** HEK‐293 T cells were transiently co‐transfected with PPRE‐Luc reporter and control Renilla vectors, then treated for 24 h under specified conditions. Pioglitazone and Rosiglitazone were used as positive controls. Cell lysates were analysed by dual‐luciferase assay. Data represent mean ± SD of relative luciferase activity (PPRE/Renilla), expressed as fold‐change vs. untreated control from three independent experiments performed in triplicate. *n* = 3 independent experiments. ****p < 0.0001.

**Table S1:** Basso–Beattie–Bresnahan scale.

**Table S2:** Full metabolite table.

**Data S1:** Supporting Information.

## Data Availability

The data that support the findings of this study are openly available in the UCSD Metabolomics Workbench at https://doi.org/10.21228/M8PV7C.
