# Supplementary material for: LPC18:0 Secreted by Exogenous Neural Stem Cells Potentiates Neurogenesis and Functional Recovery via GPR55‐Mediated Signalling in Spinal Cord Injury
Source: Cell Prolif. 2025 Nov 16;59(6):e70146. doi: 10.1111/cpr.70146 (PMC13241829; doi:10.1111/cpr.70146)
Supplement: Supplementary file 1 — Figure S1: Three‐dimensional principal component analysis (PCA) score plot of spinal cord metabolomes from sham (n = 9), SCI (n = 10), scaffold‐only (n = 8) and NSCs/scaffold (n = 9) groups. The metabolomic data acquired by LC–MS. Left: positive ion mode; Right: negative ion mode. Figure S2: Identification of Sphingosine and LPC18:0 (A) Representative MS/MS spectra of sphingosine (precursor ion *m/z* 300.29) acquired from standard (top) or tissue samples (bottom). Characteristic fragment ions at *m/z* 252.26, 264.26 and 282.27 were observed. (B) Representative MS/MS spectra of LPC18:0 (precursor ion *m/z* 524.38) acquired from standard (top) or tissue samples (bottom). A characteristic fragment ion at *m/z* 184.07 was observed. Figure S3: (A) Concentration‐dependent cytotoxicity of LPC18:0 on neural stem cells after 24 h of treatment (n = 3 independent experiments). Statistical significance was determined by one‐way ANOVA with Tukey's post hoc test compared to the control group. In vitro viability began to decrease at 20 μM. (B) The range of in vivo doses (0–30 μM) was chosen to establish a complete dose–response relationship. This dosing regimen was confirmed to be well‐tolerated in our SCI model. The in vivo doses (0–30 μM) were selected to establish a complete dose–response relationship. All doses were well‐tolerated in the animals, with no signs of systemic toxicity observed throughout the study. The BBB scores were assessed at 10 weeks after SCI following LPC 18:0 treatment at the indicated doses (n = 6 per group). Data represent mean ± SD. Statistical significance was determined by one‐way ANOVA with Tukey's post hoc test. *p < 0.05, **p < 0.01, ***p < 0.001 and ****p < 0.0001. Figure S4: HEK‐293 T cells were transiently co‐transfected with PPRE‐Luc reporter and control Renilla vectors, then treated for 24 h under specified conditions. Pioglitazone and Rosiglitazone were used as positive controls. Cell lysates were analysed by dual‐luciferase assay. Data repre [file CPR-59-e70146-s002.docx]

**Supplemental information**

**Supplemental Figure1**


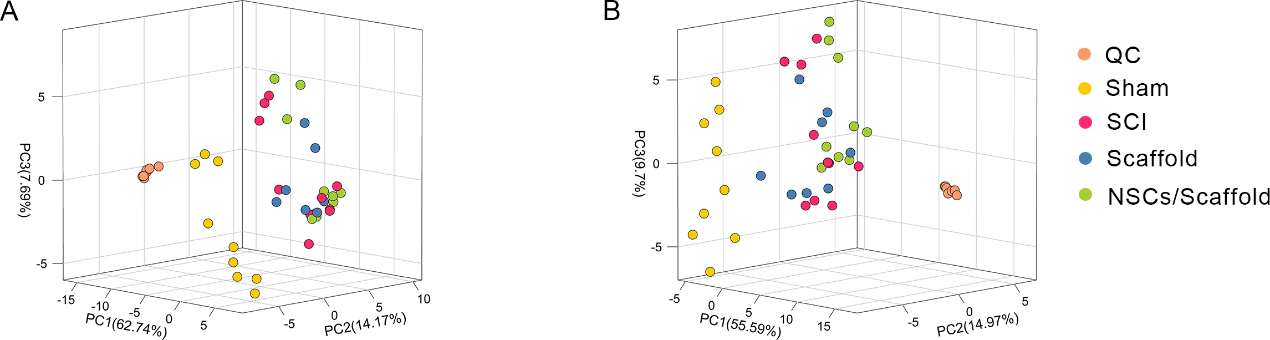


**Supplemental Figure1:** Three-dimensional principal component analysis (PCA) score plot of spinal cord metabolomes from sham (n = 9), SCI (n = 10), scaffold-only (n = 8) and NSCs/Scaffold (n = 9) groups. The metabolomic data acquired by LC-MS. Left: positive ion mode; Right: negative ion mode.

**Supplemental Figure2**


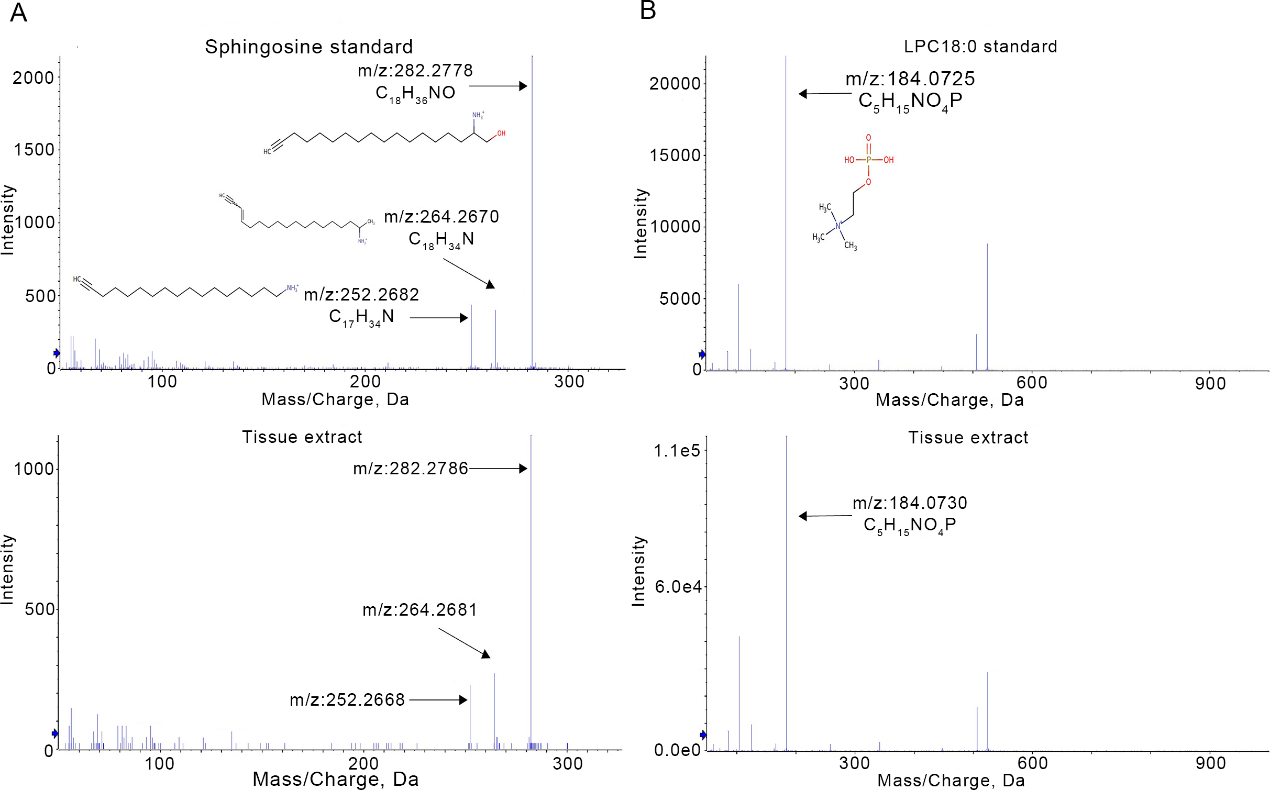


**Supplementary Figure 2: Identification of Sphingosine and LPC18:0** **(A)** Representative MS/MS spectra of sphingosine (precursor ion *m/z* 300.29) acquired from standard (top) or tissue samples (bottom). Characteristic fragment ions at *m/z* 252.26, 264.26, and 282.27 were observed. **(B)** Representative MS/MS spectra of LPC18:0 (precursor ion *m/z* 524.38) acquired from standard (top) or tissue samples (bottom). A characteristic fragment ion at *m/z* 184.07 was observed.

**Supplemental Figure3**


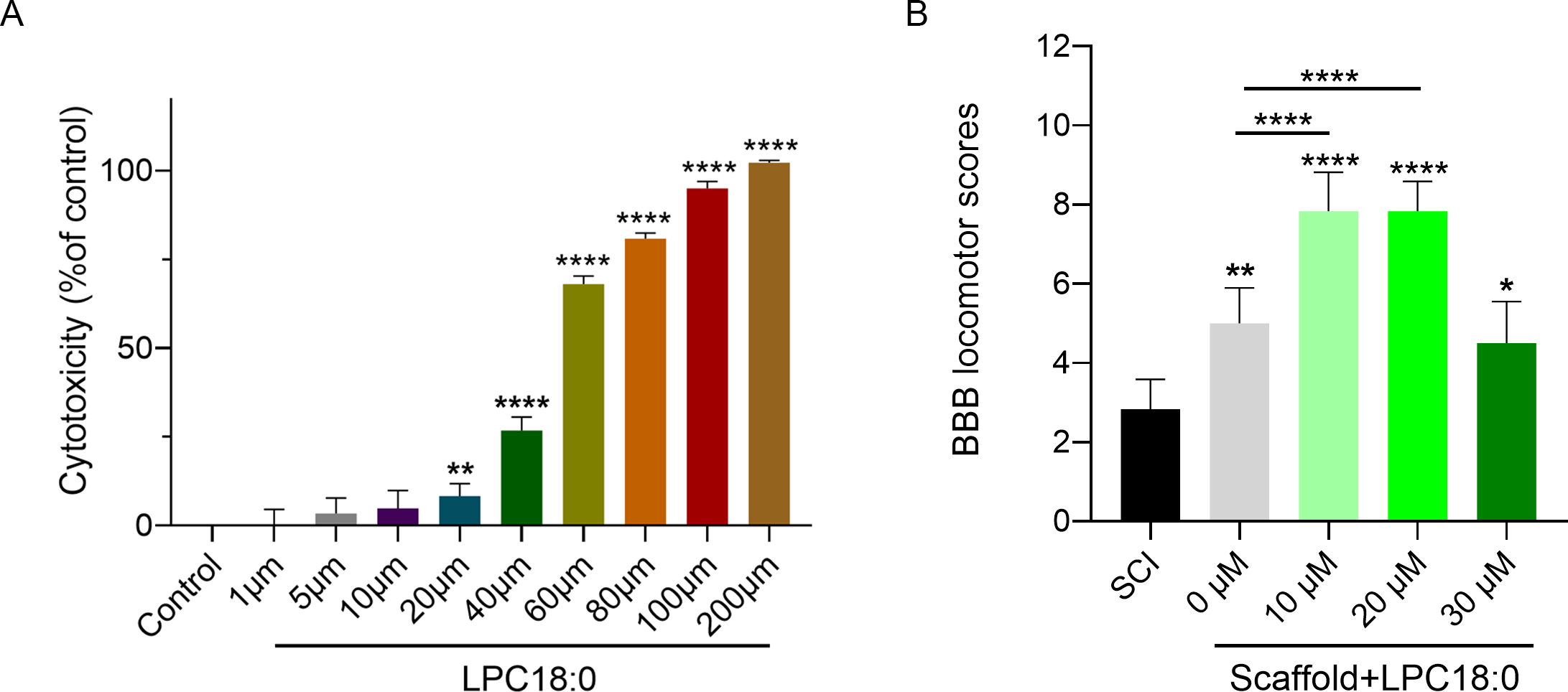


**Supplemental Figure3:** (A) Concentration-dependent cytotoxicity of LPC18:0 on neural stem cells after 24 hours of treatment (n = 3 independent experiments). Statistical significance was determined by one-way ANOVA with Tukey's post-hoc test compared to the control group. In vitro viability began to decrease at 20 μM. (B) The range of *in vivo* doses (0-30μM) was chosen to establish a complete dose-response relationship. This dosing regimen was confirmed to be well-tolerated in our SCI model. The *in vivo* doses (0-30 μM) were selected to establish a complete dose-response relationship. All doses were well-tolerated in the animals, with no signs of systemic toxicity observed throughout the study. The BBB scores were assessed at 10 weeks after SCI following LPC 18:0 treatment at the indicated doses (n = 6 per group). Data represent mean ± SD. Statistical significance was determined by one-way ANOVA with Tukey's post-hoc test. *p < 0.05, **p < 0.01, ***p < 0.001, ****p< 0.0001.

**Supplemental Figure4**


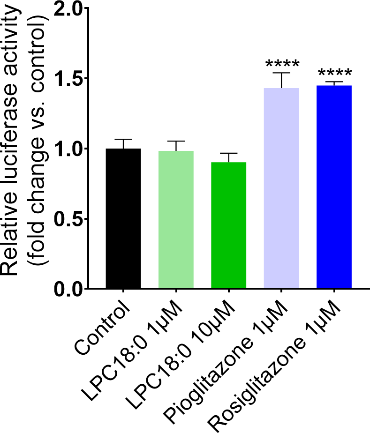


**Supplementary Figure 4:** HEK-293T cells were transiently co-transfected with PPRE-Luc reporter and control renilla vectors, then treated for 24 h under specified conditions. Piogolitazone and Rosiglitazone were used as positive controls. Cell lysates were analyzed by dual-luciferase assay. Data represent mean ± SD of relative luciferase activity (PPRE /Renilla), expressed as fold-change versus untreated control from three independent experiments performed in triplicate. n = 3 independent experiments. *****p* < 0.0001.
