# Supplementary material for: LPC18:0 Secreted by Exogenous Neural Stem Cells Potentiates Neurogenesis and Functional Recovery via GPR55‐Mediated Signalling in Spinal Cord Injury
Source: Cell Prolif. 2025 Nov 16;59(6):e70146. doi: 10.1111/cpr.70146 (PMC13241829; doi:10.1111/cpr.70146)

**LPC18:0 Secreted by Exogenous Neural Stem Cells Potentiates Neurogenesis and Functional Recovery via GPR55-Mediated Signalling in Spinal Cord Injury**

**Blots of figure 6C**


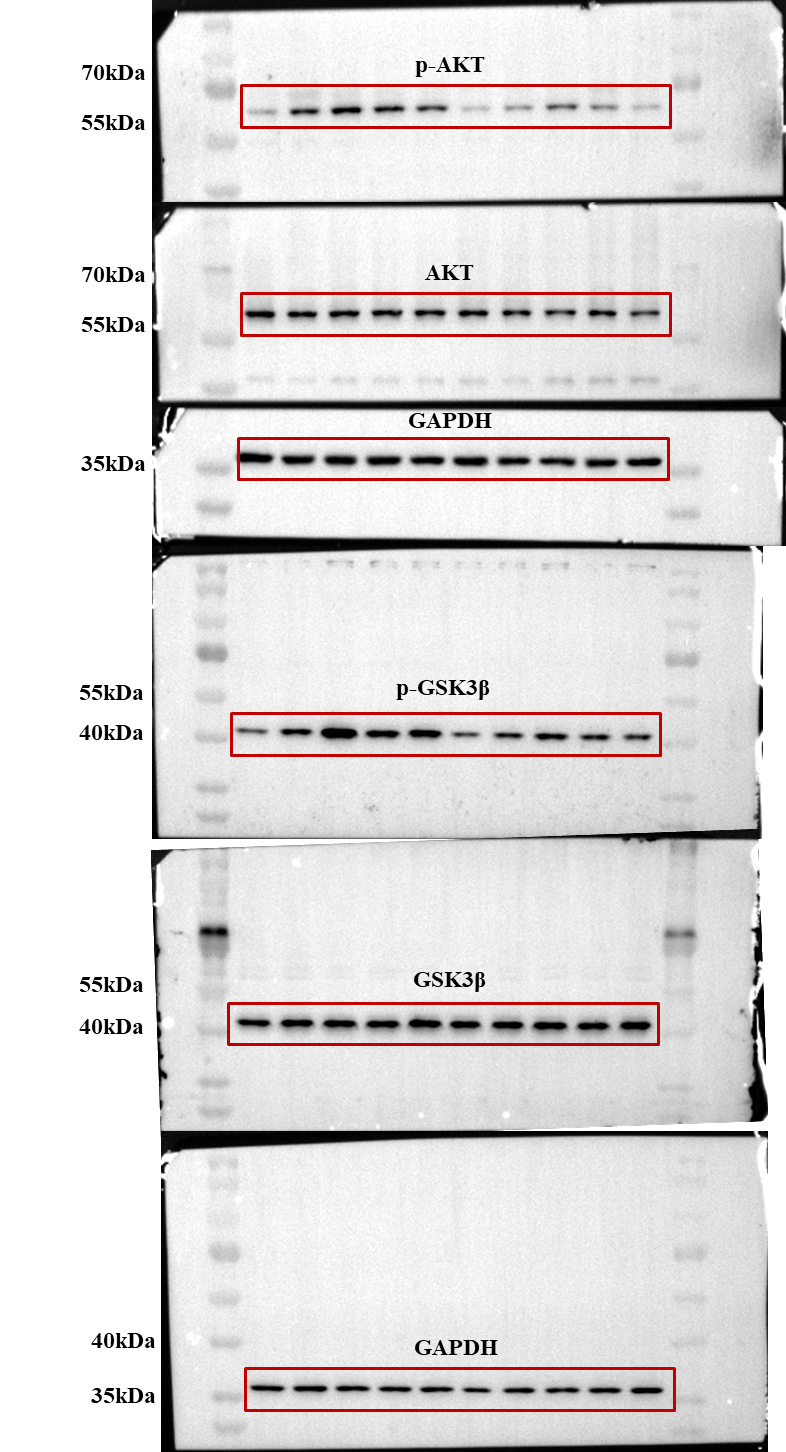


**Blots of figure 6D**


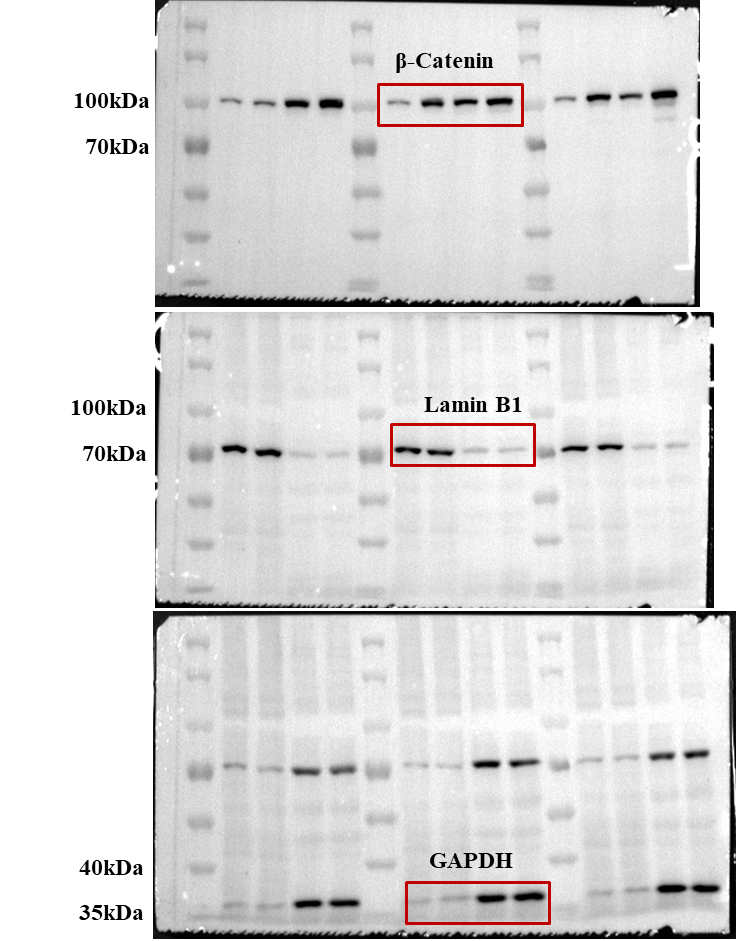

Supplement: Supplementary file 4 — Data S1: Supporting Information. [file CPR-59-e70146-s003.docx]
